# The use of 0.5r_cav_ as an effective point of measurement for cylindrical chambers may result in a systematic shift of electron percentage depth doses

**DOI:** 10.1002/acm2.12797

**Published:** 2020-01-03

**Authors:** Princess C. Anusionwu, Jorge E. Alpuche Aviles, Stephen Pistorius

**Affiliations:** ^1^ CancerCare Manitoba Winnipeg Canada; ^2^ Department of Physics & Astronomy University of Manitoba Winnipeg Canada; ^3^ Department of Radiology University of Manitoba Winnipeg Canada

**Keywords:** dosimetry protocols, electrons, EPOM, PDD, radius of cavity (*r_cav_)*

## Abstract

Electron dosimetry can be performed using cylindrical chambers, plane‐parallel chambers, and diode detectors. The finite volume of these detectors results in a displacement effect which is taken into account using an effective point of measurement (EPOM). Dosimetry protocols have recommended a shift of 0.5 r_cav_ for cylindrical chambers; however, various studies have shown that the optimal shift may deviate from this recommended value. This study investigated the effect that the selection of EPOM shift for cylindrical chamber has on percentage depth dose (PDD) curves. Depth dose curves were measured in a water phantom for electron beams with energies ranging from 6 to 18 MeV. The detectors investigated were of three different types: diodes (Diode‐E PTW 60017 and SFD IBA), cylindrical (Semiflex PTW 31010, PinPoint PTW 31015, and A12 Exradin), and parallel plate ionization chambers (Advanced Markus PTW 34045 and Markus PTW 23343). Depth dose curves measured with Diode‐E and Advanced Markus agreed within 0.2 mm at R_50_ except for 18 MeV and extremely large field size. The PDDs measured with the Semiflex chamber and Exradin A12 were about 1.1 mm (with respect to the Advanced Markus chamber) shallower than those measured with the other detectors using a 0.5 r_cav_ shift. The difference between the PDDs decreased when a Pinpoint chamber, with a smaller cavity radius, was used. Agreement improved at lower energies, with the use of previously published EPOM corrections (0.3 r_cav_). Therefore, the use of 0.5 r_cav_ as an EPOM may result in a systematic shift of the therapeutic portion of the PDD (distances < R_90_). Our results suggest that a 0.1 r_cav_ shift is more appropriate for one chamber model (Semiflex PTW 31010).

## 1 INTRODUCTION

Electron percentage depth dose (PDD) curves are commonly measured for commissioning and quality assurance of electron beams. The PDD curves can be measured using a variety of detectors, each with unique response characteristics and limitations, potentially leading to variations in the data acquired.^1^, ^2^ Different types of detectors are recommended by various dosimetry protocols for particular measurement situations. Cylindrical ionization chambers, a commonly used detector, are recommended by the AAPM TG‐25^3^ and TG‐70^1^ protocols. Cylindrical chambers have not been typically recommended for electron energies below 10 MeV. While new works suggests that their use on these small energies may not be incorrect,^4^ the use of parallel plate chambers is still recommended for the most part.[3, 5, 6, 8]

In radiation therapy, the dose to the medium rather than the detector's sensitive volume is required. Since the chamber wall and air cavity displace a volume of the media, which in turn affects the electron fluence in the cavity, the dose to water measured at the reference point of an ionization chamber differs from the dose in the absence of the detector. Thus, it is necessary to apply a correction factor to the raw measurements to account for the detector perturbation.^9^, ^10^ The ionization gradient at the point of measurement in an electron beam can be accounted for by applying a gradient correction factor, P_gr_. The P_gr_ of a cylindrical chamber is a function of the chamber's cavity radius and is unity for a parallel plate chamber.^1^ A second method is by positioning the chamber with its geometric center displaced from the point of measurement by an amount that offsets the effect. This point is referred to as the effective point of measurement (EPOM) — the depth in the medium where the average energy is the same as in the chamber and is usually a shift from the chamber’s reference point.^7^

The correct choice of the EPOM is particularly important when measuring depth dose curves. Thus, various studies and protocols have proposed different points within the chamber as the correct EPOM of ionization chambers.^11^, ^12^, ^13^, ^14^, ^15^ The International Atomic Energy Agency (IAEA) protocol^5^ on electron dosimetry and the American Association of Physicists in Medicine (AAPM) Task Group Reports 70 and 51 recommend a shift of 0.5*r_cav_* when using a cylindrical chamber.^1^, ^6^ This recommendation was based on studies performed by Johansson et al.^16^ and Khan^17^ to determine the magnitude of the displacement required to account for the gradient effect. On the other hand, the Institute of Physics and Engineering in Medicine (IPEM) recommends a shift of 0.6*r_cav_*.^7^ Other independent studies, using both experimental measurements and Monte Carlo based calculations, have been performed to determine the magnitude of the EPOM shift. Indra et al.^12^ reported that the EPOM shift is applied in the upstream direction from the central axis of the chamber and it varies from 0.9*r_cav_* to 0.5*r_cav_* between 6 and 20 MeV beams, respectively. Note that the word upstream will be used in this manuscript to indicate shifts in the direction toward the electron source. Similarly, the word downstream will be used to refer to shifts in the direction away from the electron source. Both upstream and downstream will be used to describe shifts applied to either PDDs or detectors. Legrand et al.^14^ experimentally concluded that the corrections recommended in the protocols for cylindrical chambers were not completely appropriate. They suggested applying an EPOM shift equal to 0.87(*r*_cav_ – 1 mm). The experimental work by Huang et al.^13^ showed that the use of a constant 0.5 *r_cav_* for all electron beams is too simplistic and that this value is expected to approach 0.8 *r_cav_* with increasing energy*_._*

Using Monte Carlo simulations, Wang and Rogers^18^ recommended a shift of 0.4 *r_cav_*–0.5 *r_cav_* for depth dose measurement using cylindrical chambers. Work by Voigts‐Rhetz et al.^15^ showed that the EPOM shift of cylindrical chambers is close to the recommended value of 0.5*r_cav_* at higher energies but decreases by over 30% at lower energies. Table 1 summarizes the EPOM shifts for cylindrical chambers reported in the literature.

**Table 1:** Summary of recommended shifts for cylindrical chambers by different authors.

Author	Recommended shift
Legrand et al.^14^	0.87 (*r_cav_* – 1 mm)
Indra^12^	0.9*r_cav_* at 6 MeV; 0.5*r_cav_* at 20 MeV
Huang et al.^13^	0.8*r_cav_* at higher energies
Wang and Rogers^18^	0.4*r_cav_*–0.5*r_cav_*
Voigts‐Rhetz et al.^15^	0.5*r_cav_* at higher energies and 0.3*r_cav_* at 6 MeV
AAPM TG 25 & TG 70 report^1^, ^3^	0.5*r_cav_*
IPEM^7^	0.6*r_cav_*

Similar studies have shown that the EPOM also varies for different types of parallel plate chambers. Voigts‐Rhetz et al.,^15^ from their study, found that the EPOM of parallel plate chambers differs from their reference point, except for the PTW Advanced Markus (PTW, Freiburg, Germany) chamber whose EPOM coincides with the reference point. Lacroix et al.^19^ found the EPOM of the NACP‐02 (IBA‐Scanditronix, Uppsala, Sweden) chamber to be between 0.4 mm for 6 MeV and 1.2 mm for 18 MeV (below the entrance window). Looe et al.^20^ experimentally determined the EPOM to be a downstream shift of 0.4 mm for the Markus and Roos chambers while Wang and Rogers^18^ reported a downstream shift of about 0.2 to 0.4 mm for plane‐parallel chambers.

These variations in EPOM shifts can lead to systematic shifts of the PDD depending on the value used. For instance, for an ion chamber with an *r_cav_* of 3 mm, the variation in EPOM shift can range from 0.9 mm (using 0.3*r_cav_*) to 2.7 mm (using 0.9*r_cav_*). This study compared the PDD curves measured with different detectors to investigate the effects of different EPOM shifts.

## 2 MATERIALS AND METHODS

### 2.1 Detectors

Cylindrical and parallel plate chambers are recommended by various dosimetry protocols^1^, ^3^, ^5^, ^7^, ^21^ for use in electron dosimetry. In addition, AAPM TG‐106^2^ recommends the electron diode detector as an ideal detector for electron dosimetry. An advantage of the diode detector is that it gives the percentage PDD curves directly, thereby eliminating the need for conversion factors. Based on these recommendations, ion chambers and electron diodes were investigated in this study. PDDs measured with chambers and diodes were compared with the PDDs from a similar detector in the same family but with different EPOM. This enabled us^1^ to justify the accuracy of the EPOM value used for the diode and parallel plate chambers and^2^ to quantify the effect that the 0.5*r_cav_* EPOM has on different cylindrical chambers.

Three cylindrical chambers were investigated in this study. The selected chambers included the PTW 31010 Semiflex (PTW, Freiburg, Germany), the Exradin A12 — a Farmer‐type chamber (Standard Imaging, Inc. Middleton, WI, USA) and the PTW 31015 PinPoint (PTW, Freiburg, Germany) chamber. The Semiflex chamber was selected because it is one of the most commonly used detectors and EPOM shifts are available in protocols and previous publications.^13^, ^14^, ^15^ The Semiflex is also currently used for routine QA in our department. The Exradin A12 and PinPoint chambers were chosen since they have larger and smaller cavity radius, respectively, than the Semiflex chamber. The Exradin A12 is a Farmer chamber and is used for absolute dosimetry at our institution while the PinPoint is used for stereotactic body radiation therapy (SBRT) dosimetry.

The PTW 23343 Markus (PTW, Freiburg, Germany) and PTW 34045 Advanced Markus (PTW, Freiburg, Germany) chambers were selected as parallel plate chambers. These chambers are not water proof, and their corresponding protective caps were used to measure PDDs in water. The PDDs measured with both chambers were compared for a 6 MeV beam and 10 × 10 cm^2^ field size.

The diodes used in this study were the PTW 60017 Diode‐E (PTW, Freiburg, Germany) and the IBA SFD (IBA‐Scanditronix, Uppsala, Sweden). The Diode‐E is a diode detector designed for use in electron dosimetry^22^ while the SFD is a micro‐size sensitive volume detector used in small field dosimetry.^23^, ^24^ The SFD is also suitable for electron dosimetry according to the manufacturer. The geometry and physical characteristics of these detectors are listed in Table 2.

**Table 2:** Geometry and physical characteristics of the detectors used in this work.^a^

Chamber type	Exradin A12	Semiflex (PTW 31010)	PinPoint (PTW 31015)	Markus (PTW 23343)	Advanced Markus (PTW 34045)	Diode‐E (PTW 60017)	SFD (IBA‐Scanditronix)
Sensitive Volume (cm^3^)	0.64	0.125	0.016	0.055	0.02	0.03	0.02
Water Proof	Yes	Yes	Yes	With protection cap	With protection cap	Yes	Yes
Cavity Radius (mm)	3.1^25^	2.75	1.45	N/A	N/A	N/A	N/A
EPOM (mm)^b^	1.6	1.4	0.7	1.7^20^	1.3^15^	1.3^26^, ^27^	0.8

a The dimensions stated here are taken from the product documentation provided by the manufacturers ^(¥; ∆)^ unless otherwise indicated.

b The EPOM of the cylindrical chambers was calculated using 0.5*r_cav_*. The EPOM of the SFD was provided by the manufacturer.

### 2.2 Data acquisition

PDDs were measured on a Varian Clinac 2100 CD Linac (Varian Medical Systems, Palo Alto, CA, USA) having electron energies of 6, 9, 12, 15, and 18 MeV. The commercial MP3 (PTW, Freiburg, Germany) automated water scanning system was used to acquire beam data, and the scanning direction was always from bottom to the surface of the water with a source‐to‐surface distance of 100 cm. The detectors were moved in steps of 0.5 mm in the build‐up region and 1 mm elsewhere except for the Diode‐E, which was sampled at 0.1 mm in the build‐up region. The integration time at each position was set to 2 s to minimize noise in the measurements. Ionization measurements were converted to PDDs following the AAPM TG 70 and AAPM TG 51 recommendation implemented in the MEPHYSTO software (PTW, Freiburg, Germany). This conversion consists of multiplying the percentage depth ionization curve by the ratio of the mass collision stopping powers for air and water and the fluence correction factor (P_fl_).^1^, ^6^ Perpendicular lateral profiles were acquired after each setup to ensure the detector was placed on the central axis. The depth at which the dose equals 50% of the maximum dose, R_50,_ was used to characterize the PDDs and evaluate the shifts as opposed to the depth of maximum dose (d_max_ or R_100_). This depth was used because beam quality in electron beams can be specified by R_50_/1, 6 In addition, the PDD curves for higher energies tend to have a broader and flatter d_max_ region, which can result in an incorrect determination of R_100_, particularly in the presence of noise. Most of the analysis in this work was performed for a 10 × 10 cm^2^ and 25 × 25 cm^2^ field size. The 10 × 10 cm^2^ was used because it is the reference field as specified in the recommendations of AAPM TG 106 report,^2^ while the 25 × 25 cm^2^ field is the largest field that is being used clinically at our institution. PDDs were also measured in the absence of an applicator by setting the machine jaws to 40 × 40 cm^2^. Measurements for a 40 × 40 cm^2^ field size are required as part of the commissioning process of a macro‐Monte‐Carlo‐based dose calculation algorithm^28^ and represent an extreme field size.

The reference points of the cylindrical chambers were positioned by eye on the water surface by adjusting its position until its reflection formed a perfect circle.^2^ The chambers were then shifted downstream by 0.5*r_cav_* to account for the EPOM correction. Additional EPOM corrections, for example, 0.3*r_cav_* downstream shift, were investigated by shifting (downstream) the PDD measured using 0.5*r_cav_* in the MEPHYSTO software (PTW, Freiburg, Germany). The validity of this approach was confirmed by measuring PDDs with the Semiflex chamber with EPOM corrections of 0.5*r_cav,_* 0.3*r_cav_* and 0.1*r_cav_*. The PDDs measured using 0.5*r_cav_* were shifted in the software by the difference of the theoretical values. The R50 values agreed with those measured with their corresponding shifts by 0.1 mm in both cases. Therefore, the only additional information that would be gained from the measurement would be related to setup uncertainties; which were quantified separately (Section 2.C). The use of 0.3*r_cav_* shift has been suggested by Voigts‐Rhetz et al.,^15^ especially at 6 MeV. The suitability of this shift was not investigated for higher energies since, according to the authors, their results at higher energies agree with the 0.5*r_cav_* suggested by current dosimetry protocols. The Farmer type (Exradin A12) and the Pinpoint chambers, which have different cavity radius and sensitive volume (see Table 2), were used to investigate the impact of the chamber cavity radius since detectors report averaged dose over their sensitive volume.^29^ Depth dose measurements with the Advanced Markus (plane‐parallel) chamber were taken with the top of the protective water cap of the chamber positioned at the surface of the water, then, the chamber was shifted upstream by 1.3 mm which corresponds to the EPOM. This corresponds to a summation of the depth of reference point of the chamber (Z_R_) and the EPOM shift (∆z) as found from literature.^15^ The depth dose curves obtained were compared to those of a similar plane‐parallel chamber (Classic Markus) which has a different EPOM (see Table 2), as a consistency check. The EPOM has also been found from literature data.^20^ The diodes were aligned parallel to the central axis of the beam and centered with their top set on the surface of the water. An upstream shift of 1.3 mm from the surface of the detector was then applied for the Diode‐E. There have not been many studies to evaluate the EPOM of diode detectors. However, a study by Underwood et al.^27^ confirmed the EPOM of the Diode‐E to be 1.33 mm as stated by the manufacturer. PDD measurements with the SFD were obtained by applying an upstream shift of 0.8 mm as recommended by the manufacturer.

### 2.3 Experimental uncertainties and statistical analysis

Measurements were repeated five times for each detector (Advanced Markus, Diode‐E, and Semiflex) on different days using a new setup each time to investigate the effect of experimental uncertainties in detector positioning and setup errors on the small shifts observed in the PDD curves. In addition, depth dose data obtained using the Semiflex were compared to readings from routine quality assurance (QA) for 3 yr also performed with the Semiflex. The routine QA readings were also used to monitor possible changes in the energy of the linac and were taken by multiple users. A test of statistical significance was performed to ensure that the shifts observed are real shifts and not due to the random nature of measurements. The Mann–Whitney Wilcoxon non‐parametric test^30^ was used because of the small sample size and to avoid the assumption that the data are normally distributed. A significance level (α) of 0.05 was used. The shifts were interpreted to be statistically significant if the *P* value is ≤ the significance level. The *P* value is a parameter in the hypothesis testing used to determine the statistical significance of the shifts observed in the detector readings.

## 3 RESULTS

### 3.1 PDDs using different detectors

**Figure 1:**
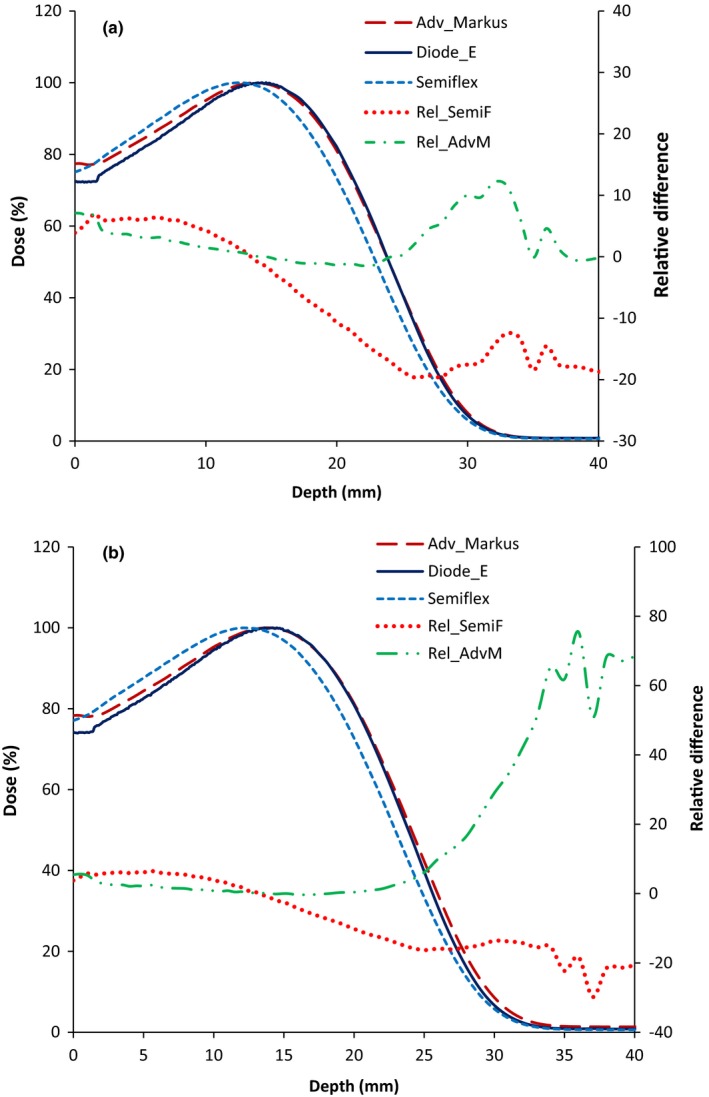
Percentage depth dose (PDD) curves for a 6 MeV electron beam measured with the Semiflex, Advanced Markus, and Diode‐E detectors. PDDs are for (a) 10 × 10 cm^2^ and (b) 25 × 25 cm^2^ electron field sizes. Relative differences with respect to the diode PDD are also shown (axis on the right side).

Figure 1 shows the depth dose curves for a 6 MeV beam measured with the Semiflex for an EPOM of 0.5*r_cav._* The figure also shows depth dose curves measured using the Advanced Markus and the Diode‐E detectors. The PDD curves shown are for a 10 × 10 cm^2^ and 25 × 25 cm^2^ electron applicators. The shapes of the depth dose curves obtained with the different detectors demonstrate the expected characteristics of an electron beam. However, the results show that there is a mismatch of the depth dose curves measured with the different detectors. More specifically, there is a systematic shift in the depth dose curve measured with the Semiflex chamber. This shift is also noticeable in the mean values of R_50_ of the three detectors obtained for various energies and field sizes as summarized in Table 3. The right axes of Fig. 1 corresponds to the relative difference for the Semiflex and Advanced Markus graphs (labeled as Rel_SemiF and Rel_AdvM, respectively) with respect to the diode. This difference was calculated as 100*(PDD_Semiflex or Markus_/PDD_Diode_ – 1). These relative difference graphs show the larger discrepancy of the PDD measured with the Semiflex.

**Table 3:** R_50_ values obtained using different detectors for different field sizes and energies.

Energy (MeV)	Field size (cm^2^)	R50 (mm)			Differences between mean R50 values (mm) of Semiflex and Advanced Markus
		Advanced Markus	Diode‐E	Semiflex	
6	10 × 10	23.9 ± 0.1	23.9 ± 0.1	22.9 ± 0.1	1.1 ± 0.1
	25 × 25	24.0 ± 0.1	24.0 ± 0.1	23.0 ± 0.1	1.0 ± 0.1
	40 × 40	24.0 ± 0.1	24.1 ± 0.1	23.1 ± 0.1	0.9 ± 0.2
9	10 × 10	36.1 ± 0.1	36.1 ± 0.1	35.0 ± 0.1	1.1 ± 0.1
	25 × 25	36.2 ± 0.1	36.3 ± 0.1	35.2 ± 0.1	1.0 ± 0.1
	40 × 40	36.3 ± 0.1	36.5 ± 0.1	35.4 ± 0.2	0.9 ± 0.2
12	10 × 10	50.2 ± 0.1	50.4 ± 0.1	49.1 ± 0.1	1.1 ± 0.1
	25 × 25	50.5 ± 0.1	50.62 ± 0.04	49.4 ± 0.1	1.1 ± 0.1
	40 × 40	50.6 ± 0.1	50.92 ± 0.04	49.6 ± 0.1	1.0 ± 0.2
15	10 × 10	63.2 ± 0.1	63.5 ± 0.1	62.1 ± 0.1	1.1 ± 0.1
	25 × 25	63.6 ± 0.1	63.9 ± 0.1	62.5 ± 0.1	1.1 ± 0.1
	40 × 40	63.9 ± 0.1	64.26 ± 0.03	62.9 ± 0.1	1.0 ± 0.2
18	10 × 10	76.0 ± 0.1	76.4 ± 0.1	74.9 ± 0.04	1.1 ± 0.2
	25 × 25	76.8 ± 0.1	77.0 ± 0.1	75.6 ± 0.1	1.1 ± 0.1
	40 × 40	77.2 ± 0.1	77.6 ± 0.1	76.1 ± 0.2	1.0 ± 0.2

As recommended by dosimetry protocols,^1^, ^2^, ^6^ the measurements from the diode detector were compared with those from an ionization chamber. The mean R_50_ values obtained from the Advanced Markus and Diode‐E curves for 10 × 10 cm^2^ field size agree within 0.3 mm 80% of time and a maximum difference of 0.4 mm. The difference between mean R_50_ values of the Semiflex and the Advanced Markus chambers is also shown in the last column of Table 3. Only the difference between the Semiflex and the Advanced Markus is reported since no statistically significant difference was found between the Advanced Markus and the Diode‐E (*P* = 1). The mean R_50_ values for the Semiflex are up to 1.1 mm smaller than those obtained with the Advanced Markus chamber for all energies and field sizes.

### 3.2 Reproducibility of experimental data

**Figure 2:**
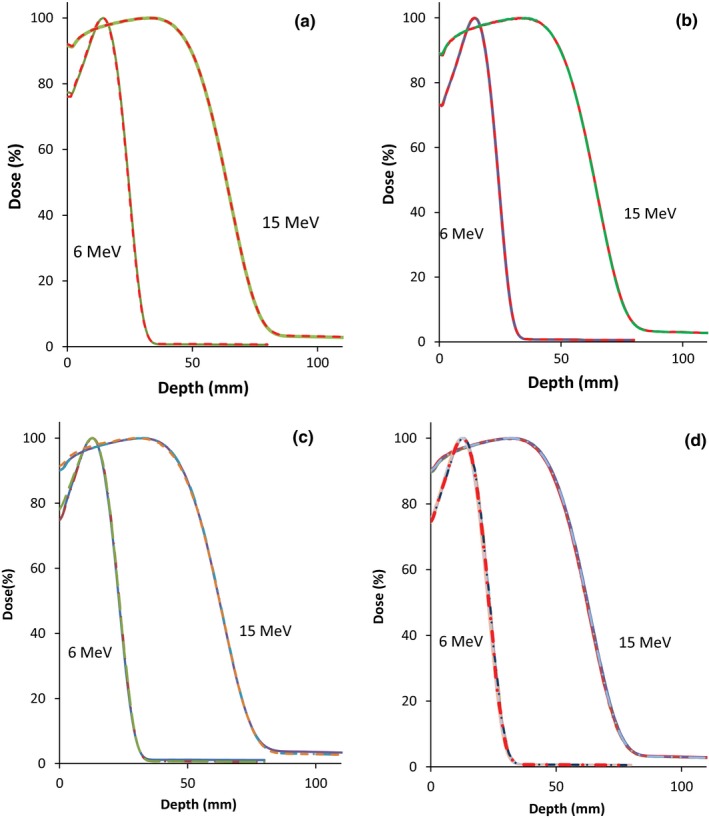
Depth dose curves from repeated measurements using (a) Advanced Markus, (b) Diode‐E, (c) Semiflex and (d) data from routine quality assurance for 6 and 15 MeV for a 10 × 10 cm^2^ field.

Figures 2(a)–2(d) shows the results of repeated measurements obtained with three detectors and routine quality assurance (QA) using the Semiflex for 3 yr (27 measurements). The results of the repeated measurements show consistency in the measurements obtained from each detector. The mean R_50_ values obtained for each detector, as shown in Table 3, agreed within ±0.2 mm. Data from routine QA with the Semiflex had an average R_50_ value of 22.9 ± 0.1 mm and 62.3 ± 0.1 mm for 6 and 15 MeV, respectively. This result agreed with the mean R_50_ values obtained from repeated measurement using a Semiflex (22.9 ± 0.1 mm and 62.1 ± 0.1 mm for 6 and 15 MeV, respectively). These results confirm that the shifts observed in the readings were not due to experimental or setup error.

In addition, a non‐parametric (Mann–Whitney Wilcoxon) test was performed to check whether the observed differences in the detector readings are statistically significant. The test showed that the difference observed between the Advanced Markus and Diode‐E was not significant (*P* = 1). On the other hand, the differences between the Semiflex and the Advanced Markus were statistically significant (*P* = 0.01). As expected, the difference between the Semiflex and the Diode‐E was also statistically significant (*P* = 0.01).

### 3.3 Investigation of the EPOM of the detectors

**Figure 3:**
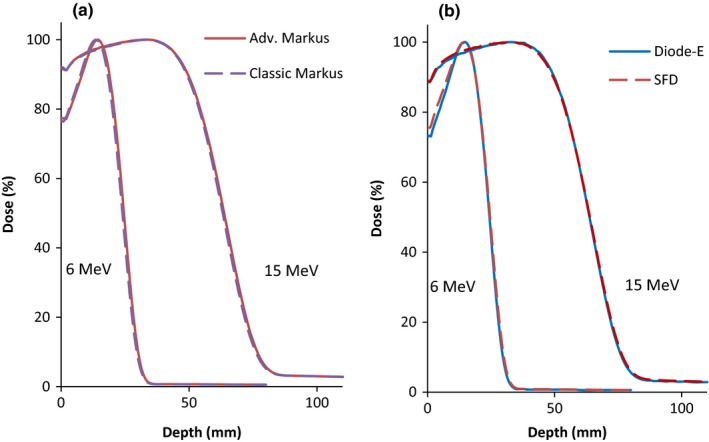
Depth dose curves measured with the (a) Classic and Advanced Markus chamber and (b) Diode‐E and SFD for 6 and 15 MeV beams using a 10 × 10 cm^2^ field.

The selection of the EPOM can result in systematic PDD shifts, and thus, it is necessary to confirm that the appropriate EPOM is used. Figure 3 shows the PDDs obtained when the detectors are compared with another detector in the same family but having a different EPOM value.

The R_50_ values of the classic and Advanced Markus chambers agreed within 0.2 mm for both 6 MeV and 15 MeV while the agreement between the Diode‐E and SFD ranged from 0.3 mm (6 MeV) to 0.2 mm (15 MeV), respectively. These agreements suggest that the shift observed in the depth dose readings was not as a result of the EPOM value used for the diode and parallel plate chamber measurements.

**Figure 4:**
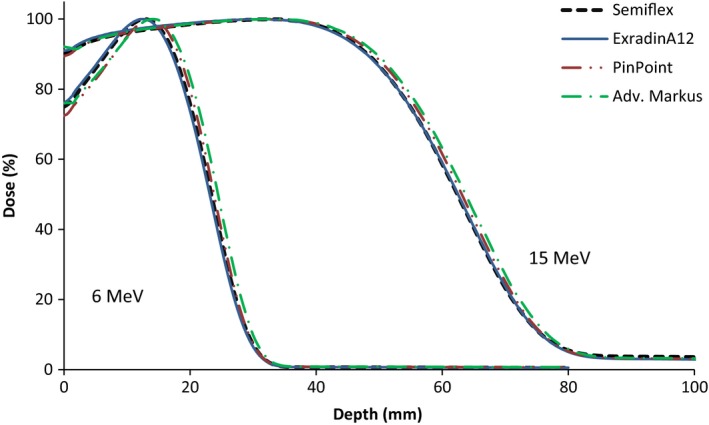
Depth dose curves at 6 and 15 MeV for a 10 × 10 cm^2^ field size using different types of cylindrical chambers.

Figure 4 shows the PDD curves obtained with the Semiflex, Exradin A12, and Pinpoint (EPOM corrections listed in Table 2). The PDD of the Advanced Markus is also shown for comparison. They verify that our observations were not only relevant to the Semiflex chamber. The R_50_ values obtained with the Semiflex and Exradin chambers, whose cavity radius are similar (see Table 2), agreed within 0.3 mm at 6 MeV and 0.1 mm at 15 MeV. This shows that both cylindrical chambers show systematic shifts and, hence, this issue is not only applicable to the Semiflex but also to other cylindrical chambers of similar cavity radius.

As the detector cavity radius and sensitive volume decrease (as with the Pinpoint), the R_50_ values tend to agree with those of the Advanced Markus and Diode‐E. This observation suggests that the larger cavity radius and sensitive volume of the Semiflex and Exradin are the reason for the disagreement observed in their PDD measurements. It is possible that this larger sensitive volume results in more significant EPOM uncertainty due to the attempt to approximate the dose deposited in a volume to a single point in space.

## 4 DISCUSSION

Measured data serve as our reference data for treatment planning. It is therefore essential to ensure that the data are of the highest quality and collected using the proper tools. Three different types of detectors, using different recommended protocols, were used to investigate how different EPOMs affected the measurement of electron PDDs using cylindrical chambers.

The PDD results showed a consistent shift between the depth dose data measured with the Semiflex and Exradin A12 when compared to those measured with the Advanced Markus and Diode‐E detectors. Measurements were repeated and compared to depth doses obtained using similar detectors to confirm this result. The results showed that the Diode‐E and Advanced Markus PDDs agreed mostly within 0.3 mm (with few exceptions) and that this difference was not statistically significant. The PDD curves measured with cylindrical chambers were systematically shallower than those measured with the other types of detectors when a 0.5*r_cav_* shift was used as an EPOM correction. The magnitude of this shift is up to 1.1 mm (with respect to the Advanced Markus chamber) and was larger than the uncertainty associated with the measurement reproducibility (standard deviation within 0.2 mm). An independent study made in parallel has recently confirmed these results.^31^ Lee et al*.* reported a difference in the range of 1.2–2.2 mm between the readings of the Semiflex and the Diode‐E for energies ranging from 6 to 16 MeV.

The magnitude of the shift is a function of the cylindrical cavity radius as demonstrated by the results obtained with the PinPoint chamber. This relationship can be explained by noting that the EPOM correction attempts to relate the dose measured in a cavity with physical size to the dose in an infinitesimally small point somewhere in that cavity. Thus, reducing the size of the cavity will lessen the uncertainty of the EPOM correction. The results from this study may be applicable to other types of cylindrical chambers. Aldosary et al. have shown that polarity and ion recombination correction are not significantly affected by changes in the wall material (differences within by 0.1% and 0.2%, respectively).^32^ Therefore, it is likely that the EPOM will be the same for cylindrical chamber with similar design but different wall materials.

**Figure 5:**
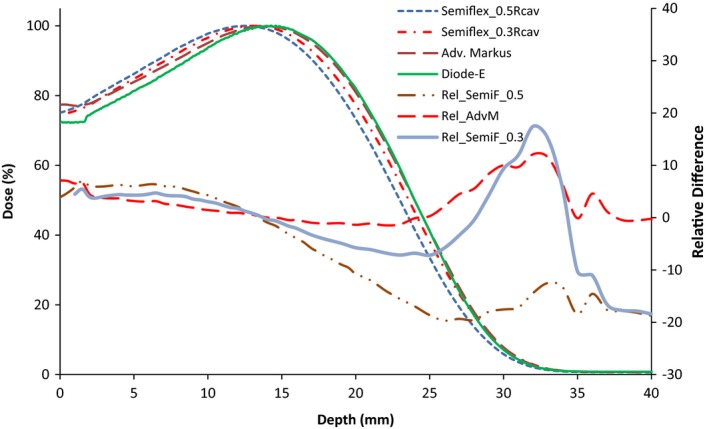
Depth dose curves at 6 MeV and 10 × 10 cm^2^ field size using a Diode‐E, Advanced Markus and Semiflex (with a downstream shift of 0.5*r_cav_* and 0.3*r_cav_*) detectors. Relative differences with respect to the diode percentage depth dose are also shown (axis on the right side).

The difference between PDDs was reduced with the use of chamber‐specific correction factors, for example, using the previously published 0.3*r_cav_* 15 for the Semiflex chamber. Figure 5 shows the result of applying an EPOM shift of 0.3*r_cav_* (as opposed to 0.5*r_cav_*) to the Semiflex chamber for the 6 MeV and a 10 × 10 cm^2^ field size. The figure shows that by applying a 0.3*r_cav_* shift, the PDD measured with the Semiflex is in closer agreement with the measurements taken with the Advanced Markus and Diode‐E chambers. The difference in R_50_ values is at most 0.5 mm when the 0.3*r_cav_* is applied. However, the 0.3*r_cav_* shift is only justified for lower energies and approaches the more common value of 0.5*r_cav_* for higher energies.^15^ This observation implies that, with a 0.3*r_cav_* shift, the use of a Semiflex chamber results in a more accurate PDD and it is, therefore, an acceptable detector for low energy electron dosimetry. However, this conclusion is inconsistent with the recommendation of the AAPM TG‐51 protocol, which states that the use of well‐guarded parallel plate chamber over cylindrical chamber is preferred for low energies (<10 MeV) but not for higher energies.

A numerical analysis of our results suggests that the Semiflex chamber was shifted by a factor of 0.4*r_cav_* more than was required. This value was obtained by dividing the 1.1 mm offset observed between the Semiflex and the Advanced Markus by the *r_cav_* of the Semiflex. Therefore, it is our finding that applying a 0.1*r_cav_*, rather than the recommended 0.5*r_cav_*, will result in the depth dose curves of the Semiflex agreeing with those of the Advanced Markus. However, it should be emphasized that this correction is only applicable for this chamber model.

**Figure 6:**
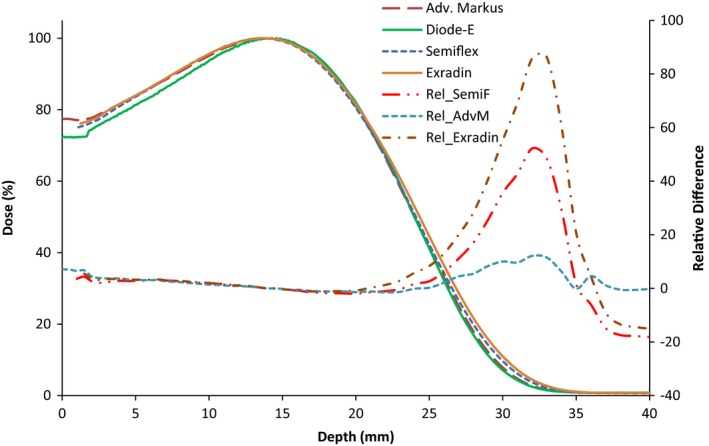
Overlap of percentage depth doses (PDDs) when a 0.1 *r_cav_* shift was applied to the Exradin A12 and Semiflex chamber measurements, relative to the PDDs for the Diode‐E and Advanced Markus chambers. Relative differences with respect to the diode PDD are also shown (axis on the right side).

The systematic PDD shifts were quantified using R_50_ as a metric. These PDD shifts were observed by overlapping the curves of different detectors by their appropriate offset. Figure 6 shows the same PDDs of Fig. 1(a) with the exception that the Semiflex curve which has been shifted by 1.1 mm (according to the last column of Table 3). Figure 6 also includes the PDD measured with the Exradin A12 chamber but shifted to mimic a PDD measured with a 0.1rcav EPOM correction. The figure shows better agreement with the other PDDs; however, the R_50_ is 0.45 mm larger than that of the Advanced Markus. This shows that the correction necessary for the Exradin A12 is different from that of the Semiflex.

Figure 6 shows the agreement of the PDDs except for two regions. The first region of disagreement is close to the surface and is anticipated given the difficulties in measuring the dose in this region. The other region of disagreement is in the descending part of the PDD. More specifically, the slope of the Diode‐E PDD is steeper than the slope of the other two curves. Attempts to match the PDDs on the descending part of the curves would result in different values of the shift. However, shifts calculated based on R_50_ are more relevant since it provides a better match in the therapeutic portion of the PDDs.

## 5 CONCLUSION

This study investigated the effect of using different EPOM values to measure electron PDDs with various detectors, particularly cylindrical chambers. It was found that the use of 0.5*r_cav_* as an EPOM results in a systematic shift of the therapeutic portion of the PDD (distances < R_90_). This shift can be as large as 1.1 mm for commonly used cylindrical chambers and decreases with a decrease in cavity size. This shift was observed for all energies and is not only of concern for low energies <10 MeV as suggested by some dosimetry protocols.^3^, ^4^, ^5^, ^6^ Our results suggest that an EPOM correction of 0.5*r_cav_* is too large and that a 0.1*r_cav_* shift gives a better agreement for a specific model (Semiflex PTW 31010).

## CONFLICT OF INTEREST

There is no conflict of interest declared in this article.
